# Occupational Demands and Workplace Resources Among Pediatric Hospital Staff During a Health System Crisis

**DOI:** 10.3390/healthcare14203448

**Published:** 2026-10-15

**Authors:** Maria Valentina Popa, Virginia Marina

**Affiliations:** 1 “Sf. Maria” Emergency Children’s Hospital, 700309 Iași, Romania; maria_valentina_popa@yahoo.com; 2 Faculty of Medicine and Pharmacy, “Dunărea de Jos” University of Galați, 800008 Galați, Romania

**Keywords:** healthcare workers, job demands–resources model, workload, organizational disruption, workplace resources, burnout prevention

## Abstract

**Background/Objectives**: The psychosocial and organizational demands of healthcare work intensify during public health crises, with implications for professional functioning. This study aimed to identify the occupational demands and workplace resources associated with professional functioning among pediatric hospital staff, using the Job Demands–Resources (JD-R) framework as the guiding theoretical model. **Methods**: A cross-sectional, mixed-methods study was conducted among 903 employees of a pediatric emergency hospital in 2024, using a custom questionnaire and thematic analysis of open-ended responses, interpreted through the JD-R framework. Multivariable logistic regression models assessed five occupational functioning outcomes, with workload, age, sex, tenure, occupational category, COVID-sector work, and relocation as predictors. **Results**: A significant workload increase was the most consistent predictor, associated with four of the five outcomes (aOR 2.04–7.16), and relocation was associated with three outcomes (aOR 1.70–2.49); COVID-sector assignment was not independently associated with the other four outcomes after correction for multiple testing, but was associated with lower odds of difficulty returning to work among infected staff (aOR 0.38). Thematic analysis identified seven themes aligned with the JD-R framework; most were significantly more frequent among COVID-sector staff after FDR correction, although a sensitivity analysis restricted to respondents who answered the open-ended item attenuated this pattern for several themes and reversed it for one. Quantitative–qualitative comparisons showed convergent associations across the constructs examined. **Conclusions**: Workload and organizational disruption were more consistently associated with impaired occupational functioning than formal sector assignment. These findings support the value of monitoring psychosocial workload indicators in occupational health surveillance and highlight team-level resources as a relevant direction for future prevention efforts.

## 1. Introduction

Healthcare work involves substantial psychosocial and organizational demands (high workload, time pressure, emotional demands, administrative burden, and work–life conflict) which consistently affect the professional functioning of healthcare staff [1,2,3].

To conceptually structure these demands and the resources available to counterbalance them, the present study uses the Job Demands–Resources (JD-R) model as its central theoretical framework [4,5]. The model posits that healthcare work environments simultaneously contain job demands, which consume energy, and job resources, which sustain engagement and reduce psychological costs [1,2,6,7]. High demands, such as workload, time pressure, emotional demands, administrative tasks, and work–life conflict, activate the health-impairment pathway, with consequences for occupational functioning [1,3,6,7,8]. Job resources, such as control over one’s work, collegial support, and teamwork, may partially buffer these effects, although the evidence is not uniform: some cohorts report consistent protective effects, while others find effects that vary by profession or are inconsistently replicated [1,9,10,11,12].

Workload remains one of the most consistently documented predictors of impaired occupational functioning among healthcare staff. In a national sample of 43,026 employees, work overload independently predicted burnout (adjusted risk ratio 2.21–2.90) and intention to leave (adjusted risk ratio 1.73–2.10) across all role types [13]. In a JD-R study among healthcare staff, workload had the strongest impact on burnout among all demands and resources examined [14]. Among nurses, higher perceived workload was associated with greater acute and chronic occupational fatigue, and among hospital auxiliary staff, psychological workload was a direct determinant of occupational fatigue [9,15]. This overload affects not only individual symptoms but also system-level functioning: time pressure and multitasking increase unit-level exhaustion, and higher workload has been linked to errors, insufficient time for care, and lower patient satisfaction [15,16,17].

Organizational change and staff relocation constitute a second, distinct category of occupational demands. Reorganizations generate uncertainty, stress, and anxiety, with negative effects on staff wellbeing [18,19], and rapid, repeated changes can produce a specific form of exhaustion (“change fatigue”) associated with professional withdrawal [20]. In pandemic contexts, role redistribution and functional changes have been associated with significant psychological distress [21], whereas staff involvement in decision-making and clear communication during change have reduced anxiety and increased acceptance of change [22].

Organizational resources play a relevant role in sustaining staff functioning during health system crises. Visible leadership and clear communication reduce anxiety and increase team cohesion [23,24], organizational and social support protect against psychological distress [25], and organizational culture and psychological support sustain long-term wellbeing [26,27]. Among intervention types tested, those focused on task modification, flexible scheduling, and changes to the work environment have the strongest empirical support [28], with psychosocial safety climate, alongside personal psychological resources, explaining a substantial proportion of variance in employee wellbeing [29]. Research on the effectiveness of organizational interventions specific to crisis contexts nonetheless remains limited [30], and employer-provided psychological support is frequently perceived as insufficient by staff [31].

These demands and resources have documented consequences for the psychological health of healthcare staff, which may include, in severe and persistent forms, burnout syndrome, recognized by the World Health Organization as an “occupational phenomenon” in the International Classification of Diseases (ICD-11) [32]. Periods of widespread public health crises substantially amplify these demands: frontline staff have reported significantly higher distress than second-line staff [33,34,35], with key organizational risk factors including workload, reduced control over work, insufficient sleep, and difficulties related to personal protective equipment [36,37,38].

The diagnostic and therapeutic complexity of pediatric emergencies, for example, the neuroimaging evaluation of acute traumatic brain injury in children [39], adds a further dimension of cognitive and decision-making demand to the activity of staff working in this type of healthcare unit, although available evidence does not consistently support more impaired functioning in pediatrics compared to other specialties [40,41], with the exception of certain subdomains such as pediatric intensive care, where repeated exposure to severe cases increases risk [42].

The cohort analyzed in this study was previously described in a publication focused on post-infectious clinical outcomes, which did not assess occupational demands, organizational factors, or available resources, aspects explicitly noted as limitations of that study [43]. Thus, particularly in pediatric settings, the associations between direct occupational demands (workload, organizational disruption through relocation) and perceived professional functioning, as well as the role of team and organizational resources in sustaining this functioning, remain insufficiently investigated.

This study aims to identify the psychosocial and organizational demands associated with occupational functioning among the staff of a pediatric emergency hospital, structured through the Job Demands–Resources framework, and to explore the team and organizational resources relevant to sustaining this functioning. The specific objectives were: (1) to assess the association between occupational demands (workload, relocation, work in a high-infection-risk sector) and indicators of professional functioning; (2) to identify, through thematic analysis of open-ended responses, the central themes of staff’s subjective experience, organized according to the JD-R framework; and (3) to explore the convergence between quantitative and qualitative data, including the possible coexistence of occupational demands with elements of professional meaning.

## 2. Materials and Methods

### 2.1. Study Design

A cross-sectional study with a mixed-methods (quantitative–qualitative) design was conducted in 2024 at the “Sf. Maria” Children’s Emergency Clinical Hospital in Iași, as part of routine occupational health examinations. The choice of this design was determined by the complementary nature of the research objectives: analyzing the relationships between occupational factors and indicators of distress required a quantitative approach, while the subjective experience of the staff was explored using qualitative methods. The two components were integrated at the level of interpretation in order to triangulate the results and obtain a more comprehensive perspective on the impact of occupational demands. Participants were asked to report retrospectively on occupational experiences relating to the COVID-19 pandemic period in Romania (from March 2020 onward), so the recall window spanned up to approximately four years before data collection in 2024.

### 2.2. Participants and the Selection Process

The target population consisted of hospital staff who worked during the COVID-19 pandemic. The study included employees who worked at the institution during the pandemic, attended their periodic occupational health checkup, and completed the questionnaire in full. Individuals who were absent from the periodic checkup, those who did not work at the hospital during the pandemic, and those who refused to participate were excluded. The final sample included 903 employees.

Data were collected using the same instrument and from the same cohort previously described by Popa et al. (2025) [43], a publication in which the post-infectious clinical outcomes of this population were analyzed. The present study addresses a distinct research question and utilizes occupational, organizational, and qualitative variables that have not been previously reported.

### 2.3. The Assessment Instrument

A custom-designed, structured questionnaire consisting of 36 items was used for data collection. It included sociodemographic and occupational information, history of SARS-CoV-2 infection, post-infection symptoms, perceived occupational impact, and an open-ended question regarding how participants were affected by the changes that occurred during the period under analysis. The instrument is not a validated questionnaire for assessing burnout. Given the heterogeneous nature of the constructs under investigation, the complete battery was not considered a unidimensional scale; consequently, an overall internal consistency coefficient was not calculated.

Two of the outcome items apply only after an infection episode: the item on night-shift capacity explicitly asks whether infection affected this capacity, and the item on returning to work concerns resuming duties after an absence. Consistent with this, among the 274 participants without a documented infection (13 further participants had unknown infection status), only one reported difficulty with night shifts and none reported difficulty returning to work; these items were therefore analyzed only among participants with a documented infection (Section 2.4).

A subset of seven symptomatic items excessive fatigue, mental exhaustion, difficulty concentrating, insomnia, stress, depression, and anxiety was used, as a sensitivity analysis (Section 3.2), in the form of a “psycho-functional symptomatic burden index.” Each symptom was rated on a four-point ordinal severity scale (0 = absent, 1 = mild, 2 = moderate, 3 = severe). The seven item scores were summed without weighting to obtain a composite score ranging from 0 to 21, with higher values indicating greater symptomatic burden. The internal consistency of this derived score was adequate (α = 0.861; item-total correlations 0.63–0.69). This coefficient reflects only the internal consistency of the index and does not constitute a validation of the instrument as a measure of burnout.

### 2.4. Variables and Quantitative Statistical Analysis

All five outcome variables were derived from single closed-ended items with binary (yes/no) response options. Each item asked participants to report, retrospectively, whether they had experienced the corresponding difficulty or symptom in relation to the occupational changes experienced during the pandemic period; responses were coded as 1 (presence) versus 0 (absence) for logistic regression modeling. The main outcome variables were defined directly based on items related to perceived occupational functioning and impact and included: severe stress, physical and mental fatigue, difficulty maintaining the pace of work, difficulty returning to work, and difficulties associated with working night shifts. Perceived change in workload was assessed using a single closed-ended item in which respondents selected one of three mutually exclusive, predefined response categories: no change, slight increase, or significant increase; this three-category format was the original response scale used in the questionnaire and was not derived by categorizing a continuous measure of workload. Explanatory variables included this workload item, age, gender, years of professional experience, occupational category, work in the COVID sector, and staff relocation or reassignment. The exact wording and coding of the items used as outcome or predictor variables were as follows: workload change (“How has your workload changed?”; no change/slight increase/significant increase; coded 0/1/2); relocation (“Were you relocated or reassigned to a different unit?”; yes/no; coded 1/0); work in the COVID sector (“Did you work in a unit designated for COVID-19 patients?”; yes/no; coded 1/0); major stress (“Did you experience major stress due to occupational changes?”; yes/no; coded 1/0); physical/mental fatigue (“Did you experience physical and mental fatigue?”; yes/no; coded 1/0); difficulty maintaining work pace (“Did you have difficulty maintaining your work pace?”; yes/no; coded 1/0); difficulty returning to work (“Did you have difficulty returning to work or performing your duties?”; yes/no; coded 1/0); difficulty with night shifts (“Did infection affect your ability to work night shifts?”; yes/no; coded 1/0); age and years of service (numeric, in years; entered as continuous variables); sex (female/male; coded 1/0); and occupational category (nurse, physician, resident physician, auxiliary care staff, administrative staff, or other; entered as a categorical variable with nurse as the reference group).

Statistical analyses were performed in SPSS, version 18.0, using a statistical significance threshold of *p* < 0.05. For each of the five outcome variables, a multivariable logistic regression model was estimated, in which all explanatory variables were included simultaneously, reporting the adjusted odds ratio (aOR) and the 95% confidence interval (95% CI). Workload was entered as a categorical variable (reference: unchanged workload), so that slight and significant increase were estimated separately rather than assuming a common per-category effect; a respondent who selected two contradictory workload categories was treated as having an invalid (missing) response. Occupational category was collapsed into three groups (nurse [reference], physician/resident physician, and other staff) because the original six-category scheme produced strata too small for stable estimation in the two models restricted to the infected subgroup (Section 2.4). For bivariate comparisons, the Mann–Whitney U and Kruskal–Wallis tests were used, and for the analysis of associations between categorical variables, the χ^2^ test and Fisher’s exact test were applied, the latter being used in situations where the expected frequencies were less than 5. For the multiple comparisons performed on the seven qualitative themes, and jointly across the fifteen primary regression coefficients of interest (significant workload increase, relocation, and COVID-sector assignment, for each of the five outcomes), the Benjamini–Hochberg correction for the false discovery rate (FDR) was applied, and both the unadjusted *p*-values and the adjusted q-values were reported.

Of the full cohort (n = 903), 122 participants (13.5%) were excluded from the multivariable models because of missing data on at least one model variable (complete-case analysis): 117 because the workload item was missing or invalid (116 respondents left it unanswered and one selected two contradictory categories) and 5 because of missing age or years of service with workload otherwise present (a sixth participant had both age/tenure and workload missing and is already counted among the 117). No missing data were observed for sex, occupational category, work in the COVID sector, or relocation status. The analytic sample was n = 781 for the models of major stress, physical/mental fatigue, and difficulty maintaining work pace. As sensitivity analyses, we additionally (i) re-estimated all five models after multiple imputation of the missing workload, age, and tenure values (20 imputed datasets, combined using Rubin’s rules; Section 4.8) and (ii) re-estimated the two infection-restricted models using Firth’s penalized likelihood, as a secondary check on the standard maximum-likelihood estimates reported as the primary analysis.

The multiple-imputation sensitivity analysis was performed under a Missing At Random (MAR) assumption, whereby missingness was assumed to be explainable by the observed variables included in the imputation models.

Difficulty returning to work and difficulty with night shifts were analyzed only among participants with a documented infection and complete covariate data (n = 562), for the reason given in Section 2.3. Even with the three-group occupational category, these two models retain a limited events-per-variable ratio (58 and 65 events for 9 parameters, respectively); the corresponding estimates, particularly their wider confidence intervals, should be interpreted with some caution.

### 2.5. Qualitative Component

The responses to the open-ended question were analyzed using an inductive thematic analysis approach, conducted collaboratively by two researchers through an iterative process of review, discussion, and consensus-building regarding the final codes and themes. No initial, completely independent coding stage was conducted, and consequently, no formal inter-rater agreement coefficient was calculated; this aspect is noted below among the study’s limitations.

Both researchers independently read the full set of responses to gain familiarity with the data prior to any coding. Initial codes were then generated collaboratively through iterative discussion, with codes progressively grouped into candidate themes and refined until consensus was reached on a final set of seven themes, each accompanied by an explicit working definition and illustrative examples. Consensus coding, while methodologically weaker than independent double-coding with formal reconciliation, is a recognized approach in applied qualitative health research, particularly for exploratory thematic analyses embedded within a primarily quantitative study.

Each response could be classified simultaneously under one or more themes; among participants who reported at least one theme, the average was 1.35 themes per respondent. Therefore, the thematic categories were not mutually exclusive, and the cumulative percentages do not total 100%. The seven emerging themes were identified inductively and subsequently framed, a posteriori, within the Job Demands–Resources theoretical framework [4,5]. The JD-R model was used exclusively as a conceptual tool for organizing and interpreting the identified themes. Coding decisions were made jointly rather than independently and then reconciled, so no formal inter-rater agreement statistic could be calculated, and thematic saturation was not formally assessed, as the open-ended question was administered once to the full sample rather than through iterative sampling; both points are noted as limitations in Section 4.8.

## 3. Results

### 3.1. Characteristics of the Cohort

The cohort analyzed included 903 employees, with a predominance of women and a majority of nursing staff. The average age of the participants was 47.76 ± 8.55 years, and the average length of service was 18.10 ± 10.34 years. Nurses constituted the largest occupational group, followed by auxiliary care staff. Approximately two-thirds of the participants reported a slight or significant increase in their workload, 28.7% were relocated or reassigned, and 28.0% worked in COVID-19 units. The sociodemographic and occupational characteristics of the cohort are presented in Table 1.

Of the 903 participants, 781 had complete data for the primary models and 122 were excluded (Section 2.4). Excluded participants had a shorter length of service (mean 14.4 vs. 18.6 years, *p* < 0.001), were less often assigned to COVID-19 units (4.9% vs. 31.6%, *p* < 0.001), differed in occupational category (*p* = 0.012), and reported lower frequencies of major stress (21.3% vs. 55.1%), physical/mental fatigue (30.3% vs. 72.5%), and difficulty maintaining work pace (0.8% vs. 16.4%) (all *p* < 0.001); age did not differ (47.5 vs. 47.8 years, *p* = 0.50). Missing data were therefore unlikely to be missing completely at random, and complete-case estimates may not generalize to the excluded participants.

### 3.2. Occupational Predictors of Distress and Functional Impairment

Work in the COVID sector was not independently associated with major stress, physical/mental fatigue, difficulty maintaining work pace, or difficulty with night shifts after FDR correction (Table 2). It was, however, associated with lower odds of difficulty returning to work among infected staff (aOR = 0.38, 95% CI 0.17–0.86, q = 0.037); this association is addressed further below and in Section 4.2. Difficulty returning.

A significant workload increase was the most consistent predictor, associated with four of the five outcomes, and relocation was associated with three outcomes. Working in the COVID sector was not an independent predictor of major stress, physical/mental fatigue, difficulty maintaining work pace, or difficulty with night shifts, but was independently associated with lower odds of difficulty returning to work among infected staff.

As a sensitivity analysis, the seven-item symptomatic burden index (each symptom was rated on a four-point ordinal severity scale: 0 = absent, 1 = mild, 2 = moderate, and 3 = severe; the seven item scores were summed without weighting to obtain a composite score ranging from 0 to 21, with higher values indicating greater symptomatic burden) was analyzed as a continuous outcome using linear regression with the same predictors as the primary models (n = 781; median = 0, mean = 1.51; R^2^ = 0.023). A significant workload increase remained associated with a higher burden score (B = 0.70, 95% CI 0.05–1.35, *p* = 0.037), consistent in direction with the binary outcomes above, whereas a slight workload increase, relocation, and COVID-sector assignment were not significant. Because the index has a pronounced zero-heavy distribution (median = 0), we additionally examined this association using heteroskedasticity-robust (HC3) standard errors (*p* = 0.056), a bootstrap 95% confidence interval (0.03–1.44), and a nonparametric Mann–Whitney U test comparing participants with a significant workload increase to those with unchanged workload (both group medians = 0; *p* = 0.306). These checks do not uniformly support the association, and we therefore interpret this analysis as directionally consistent with, but not independent statistical confirmation of, the workload association.

Multiple imputation of missing workload, age, and tenure data (Section 2.4) preserved the direction of all principal associations; the strongest associations (significant workload increase with difficulty maintaining work pace, returning to work, and night-shift difficulty, and relocation with four of the five outcomes) remained clearly significant, while the workload association with physical/mental fatigue attenuated to borderline (*p* = 0.063) and COVID-sector assignment for physical/mental fatigue, non-significant in the complete-case model, became borderline in a positive direction (aOR = 1.55, *p* = 0.056). Firth penalized regression for the two infection-restricted outcomes produced estimates close to the standard maximum-likelihood models reported above, including for the COVID-sector association with difficulty returning to work (Firth aOR = 0.40, *p* = 0.021).

### 3.3. Emerging Qualitative Themes

Analysis of the responses to the open-ended question revealed a significant diversity in the experiences reported by staff in the context of occupational changes. Of the 903 participants, 495 (54.8%) mentioned at least one relevant theme, and the same response could be classified into multiple thematic categories simultaneously (mean 1.35 themes per respondent among those reporting at least one theme); therefore, percentages are not mutually exclusive and do not sum to 100%. A response was coded as containing no identifiable theme (408/903, 45.2%) when the participant did not answer the open-ended item; this proportion differed markedly by sector, with 23/253 (9.1%) non-respondents among COVID-sector staff compared with 385/650 (59.2%) among the remaining staff, i.e., 90.9% vs. 40.8% providing at least one identifiable theme. This differential engagement is examined directly in a sensitivity analysis below (Section 3.5) and discussed in Section 4.3. The most frequently identified theme was emotional stress and exhaustion, followed by fear of infection and concern for family safety, difficulties adapting to new roles, and professional overload. Themes related to occupational resources—namely, peer support and job satisfaction—were reported less frequently. The distribution of the seven emerging themes, subsequently organized according to the Job Demands–Resources framework, is presented in Table 3.

### 3.4. Frequency of Themes: COVID-19 Unit Staff Versus Remaining Staff

To assess whether the reported experiences differed based on direct occupational exposure, the frequency of the seven themes was compared between staff who worked in COVID-related sectors and the rest of the employees. All seven themes were reported more frequently by staff in the COVID sectors in this full-sample analysis, and the associations remained significant after Benjamini-Hochberg correction for multiple testing; however, as detailed below, this pattern was not uniformly robust to restricting the comparison to respondents who answered the open-ended item. The strongest association was observed for issues related to personal protective equipment, followed by professional fulfillment, difficulties adapting to new roles, and peer support. The magnitude of the associations, expressed as OR and 95% CI, is presented in Table 4.

### 3.5. Sensitivity Analysis: Theme Frequency Among Respondents to the Open-Ended Item

Because response engagement differed markedly by sector (Section 3.3), we repeated the comparison in Table 4 restricted to the 495 participants who answered the open-ended item (230 COVID-sector; 265 remaining staff), excluding the 408 non-respondents. The results, shown in Table 5, differ materially from the full-sample analysis.

The three themes most specifically tied to direct COVID-sector exposure (PPE-related issues, professional fulfillment, and adaptation to new roles) remained significantly more frequent among COVID-sector staff, attenuated relative to the full-sample estimates but consistent in direction. Workload/overwork and fear of infection were no longer significantly associated with sector once restricted to respondents, and emotional stress/exhaustion reversed direction, becoming significantly less frequent among COVID-sector staff in this subgroup (OR = 0.44). This indicates that part of the full-sample between-sector differences for these three themes, most clearly for emotional stress/exhaustion, reflected differential response engagement rather than a difference in underlying experience, and we interpret the full-sample estimates for these three themes with corresponding caution (Section 4.3).

### 3.6. Convergence Between Quantitative and Qualitative Data

To assess the convergence between the quantitative and qualitative components of the study, pairs of variables describing comparable occupational constructs were analyzed. A significant increase in workload was associated with spontaneous mentions of being overworked in the open-ended responses, while staff reassignment was associated with the theme of adapting to new roles. Work in the COVID sector showed the strongest association with reports of problems related to personal protective equipment, while high levels of stress reported in the quantitative component were associated with the qualitative theme of emotional stress and exhaustion. All four relationships analyzed were statistically significant, supporting the convergence between the structured data and the data obtained from the open-ended responses. The results are presented in Table 6. For the workload pair, the 117 participants with missing or invalid workload data were excluded (n = 786).

### 3.7. Integration of Quantitative and Qualitative Findings

The integration of quantitative and qualitative components allowed us to contextualize the statistical associations through the experiences reported directly by the participants. Work overload, adaptation to new roles, and difficulties associated with personal protective equipment showed a marked convergence between the two components of the study. At the same time, peer support and a sense of professional usefulness emerged as perceived resources, reported particularly in the context of intense occupational exposure. The integration of these results into the Job Demands–Resources framework is summarized in Table 7.

## 4. Discussion

### 4.1. Workload and Organizational Disruption as Dominant Risk Factors

The results of this study indicate that perceived workload and staff relocation/redistribution are the occupational determinants with the most consistent and statistically robust association with impaired professional functioning, as assessed by the five indicators analyzed. Workload remained significant, after multivariable adjustment, for four of the five outcome variables, with a notable gradient of association for difficulty in maintaining work pace (aOR = 7.16 for a significant increase and 1.98 for a slight increase, relative to unchanged workload), with a graded pattern across outcomes, showing a graded association between the perceived intensity of demand and self-reported functional capacity. This finding is supported by the literature, which consistently highlights work overload as a major factor associated with occupational distress among healthcare personnel, with adjusted risks reported in the international literature ranging from 2.21 to 2.90 [13], and with recent reviews that include workload and extended work schedules among the main organizational determinants of professional burnout during health crises [36,44].

From the perspective of occupational medicine, this finding has a direct practical implication: unlike exposure to infectious agents, which is difficult to modify structurally in the context of a public health crisis, workload and the way work is organized are organizational variables that the management of a healthcare institution can directly influence—a premise supported by evidence from direct interventions, which show that changes in the work environment and work organization can measurably reduce the prevalence of burnout in high-demand settings [45].

From a biological standpoint, these behavioral associations are supported by evidence regarding the neuroendocrine mechanisms involved in burnout: circadian rhythm disruption and suppression of melatonin and cortisol secretion have been consistently documented among healthcare staff affected by occupational exhaustion, suggesting a bidirectional relationship between disorganized work schedules—particularly night shifts—and dysregulation of the biological clock [46,47]. Exposure to artificial blue light, common in hospital settings during night shifts, has likewise been associated with disruption of natural circadian rhythms [48], representing an additional environmental factor that may contribute to the specific vulnerability of night-shift staff. This perspective provides further physiological grounding for the practical relevance of monitoring night-shift work capacity as a potentially relevant marker of occupational strain.

### 4.2. The Role of Organizational Disruption: Relocation as a Stressor Distinct from Infectious Exposure

The finding that staff relocation or redeployment remained independently and statistically significantly associated with three of the five outcome variables, while work in the COVID sector did not show independent significant associations with four of the five analyzed indicators after correction for multiple testing (the exception, difficulty returning to work among infected staff, is discussed below), suggests the existence of distinct mechanisms linking exposure to infectious risk and organizational disruption of professional activity. This distinction is consistent with the Job Demands–Resources model [4], which distinguishes between challenge demands associated with increased professional effort, but which may retain a certain degree of predictability and professional significance, and hindrance demands, characterized by role ambiguity, unpredictability, and disruption of professional routines—the latter being more consistently associated with occupational burnout and the intention to withdraw from work [49]. In this context, relocation can be interpreted as an organizational demand with hindrance potential, as it alters the work environment, job responsibilities, and familiar professional reference points, which could explain its persistent association with indicators of distress.

In contrast, working in a COVID sector may be perceived by some employees as a professional responsibility that is more clearly defined in terms of their role, without necessarily entailing the same degree of organizational disruption. This interpretation is consistent with data highlighting the role of reduced control over work and organizational unpredictability as distinct risk factors, beyond mere occupational risk exposure [37,38]. An exception to this general pattern is discussed in Section 4.2 below: COVID-sector assignment was independently associated with lower odds of difficulty returning to work among infected staff, an association we interpret cautiously rather than as evidence of a protective causal effect.

The absence of an independent association for COVID-sector assignment with four of the five outcomes should not be read as evidence that sector assignment is unimportant. COVID-sector staff were considerably more likely to report a significant workload increase and relocation than other staff, so part of any effect of sector assignment on these four outcomes may operate indirectly through these two demands rather than independently of them; this pattern is compatible with mediation, which our cross-sectional data cannot formally test. For the fifth outcome, difficulty returning to work, COVID-sector assignment remained independently associated with lower odds among infected staff after adjustment for workload and relocation (aOR = 0.38, 95% CI 0.17–0.86, q = 0.037), and this association persisted under both multiple imputation (aOR = 0.41, *p* = 0.032) and Firth penalized regression (aOR = 0.40, *p* = 0.021), indicating that it is not an artifact of complete-case analysis or sparse-data estimation. We interpret this finding cautiously and explicitly avoid any causal or protective interpretation. Two non-exclusive explanations, which our cross-sectional design cannot adjudicate between, seem plausible: COVID-designated units may have had more structured or expedited return-to-work procedures following infection-related absence, or staff with greater baseline functional capacity may have been preferentially assigned to or retained in these units. We present this result as hypothesis-generating and recommend that future longitudinal research examine return-to-work pathways specifically.

### 4.3. Quantitative–Qualitative Integration and Convergence of Results in Mixed-Methods Design

A formal analysis of the four pairs of variables selected to assess convergence revealed consistent associations between the quantitative and qualitative components of the study. The strongest association was observed between work performed in the COVID sector and the qualitative theme regarding discomfort associated with the use of personal protective equipment (OR = 140.41; 95% CI 19.24–1024.91). This estimate should be interpreted with caution, given the very small number of cases in the reference category, a situation that resulted in a very wide confidence interval. It should also be noted that some of the tested factor–theme pairs—particularly the association between self-reported workload increase and the qualitative theme of work overload—involve conceptually proximate constructs, such that a degree of convergence might be expected by construction rather than representing fully independent corroboration; associations involving less conceptually overlapping constructs, such as general perceived stress and the broader theme of emotional exhaustion, may therefore be somewhat less affected by this overlap; even so, because all pairs originate from the same questionnaire, the findings represent descriptive convergence rather than independent validation.

A further consideration concerns the full-sample comparison of theme frequency by sector (Table 4), which used all 903 participants as the denominator, including the 408 who did not answer the open-ended item; response engagement differed markedly by sector (90.9% among COVID-sector staff vs. 40.8% among remaining staff; Section 3.3). When this comparison was restricted to the 495 respondents who answered the item (Table 5), the three themes most specifically tied to direct COVID-sector exposure (PPE-related issues, professional fulfillment, and adaptation to new roles) remained significantly more frequent among COVID-sector staff, but workload/overwork and fear of infection no longer differed significantly by sector, and emotional stress/exhaustion reversed direction, becoming significantly less frequent among COVID-sector staff in this subgroup. We therefore interpret the full-sample excess of emotional stress/exhaustion, fear of infection, and workload/overwork among COVID-sector staff as substantially confounded by differential response engagement rather than as evidence of a genuinely higher frequency of these experiences, whereas the associations for PPE-related issues, professional fulfillment, and adaptation to new roles appear more robust to this source of bias.

It is noteworthy that the strongest association was identified for a predominantly physical and sensory occupational stressor, closely linked to direct exposure in the COVID sector, in contrast to general indicators of psychological distress, for which work in the COVID sector did not show significant independent associations in the multivariable models, with the exception of difficulty returning to work among infected staff (Section 4.2). This distinction is consistent with the literature, which describes the use of personal protective equipment as a distinct occupational stressor with a predominantly physical and sensory impact, assessed separately from general psychosocial factors in studies of frontline healthcare workers [44].

Overall, these results suggest that the effects of sectoral exposure on professional performance may be specific to certain concrete job demands rather than generalized, a finding that is relevant for directing organizational interventions toward directly modifiable risk factors.

### 4.4. Qualitatively Identified Occupational Resources:Peer Support and a Sense of Professional Purpose

The thematic analysis identified two themes conceptually framed as occupational resources collegial support and professional satisfaction/sense of duty—both of which were reported significantly more frequently by staff in the COVID sector. This finding is consistent with the literature on peer support as a resource with solid empirical support for the professional resilience of healthcare workers: in a study of 2032 physicians in Sweden, each increase in the perceived level of peer support was associated with an approximately 39% reduction in the risk of severe burnout (OR = 0.61) [50], and a regional systematic review identified collegial relationships, along with decision-making autonomy and support from leadership, as the main documented protective factors among healthcare personnel [51]. Peer support and a sense of professional duty are, in fact, among the pillars of occupational resilience consistently identified in a comprehensive qualitative synthesis of the literature on healthcare workers during pandemics [52], although the same source draws attention to the risk that excessive assumption of responsibility, in the absence of adequate organizational support, may paradoxically function as an additional source of moral distress, rather than merely as a protective factor [53].

This study did not quantitatively measure these resources nor did it statistically test for any protective or moderating effects they might have. Consequently, peer support and job satisfaction are described strictly as “perceived workplace resources,” and it cannot be asserted, based on the available data, that they actually function as protective factors in a statistical sense a distinction that reflects a fundamental requirement of epistemic rigor, which is all the more important given that the literature itself documents complex, sometimes counterintuitive associations between perceived resources and burnout under conditions of chronic overload [12].

### 4.5. The Coexistence of Occupational Demands and a Sense of Professional Worth

A distinct finding of the qualitative analysis was the significantly higher frequency of the theme related to job satisfaction (T7) among staff exposed to the most intense occupational demands (OR = 17.55; *p* < 0.001). Although the absolute number of participants who expressed this theme was small, the observed association is consistent with the emerging literature describing the possibility of occupational distress coexisting with perceptions of meaning, usefulness, and professional satisfaction among healthcare workers exposed to particularly demanding work conditions. The conceptual framework of Professional Quality of Life reflects this duality through the simultaneous presence, in the same professionals, of compassion fatigue and secondary traumatic stress, alongside compassion-based satisfaction. In this context, positive correlations have been reported between perceived occupational stress and these dimensions of professional experience (r = 0.461 and r = 0.596, respectively) [54,55].

However, this finding must be interpreted with caution, given the cross-sectional and descriptive nature of the data. The results do not allow for the establishment of a causal relationship between the intensity of job demands and an increased sense of meaning or job satisfaction. They merely indicate that, among a small subgroup of participants, the experience of intense job demands coexisted with expressions of a sense of usefulness, professional duty, and satisfaction associated with the role they performed. This descriptive association supports the need for future studies to systematically investigate how occupational distress and positive professional resources can coexist in high-demand work contexts.

### 4.6. Interpretation from the Perspective of Occupational Medicine

From the perspective of occupational medicine, the results support orienting occupational surveillance beyond the simple administrative criterion of assignment to a specific sector of activity, toward a more direct assessment of perceived organizational demands—particularly workload and changes in role, structure, or work environment resulting from staff relocation and redeployment. These variables are particularly relevant because they are, at least in part, subject to managerial and organizational intervention, unlike exposure to infectious risks, which may be inherent to medical work in the context of a public health crisis [36,37].

Based on the available data, the recommendation to use a specific burnout screening tool cannot be supported, as the present study did not use or validate such an assessment method. However, the results justify the inclusion, as part of periodic occupational health monitoring, of exploratory questions regarding perceptions of workload, recent organizational changes, and any associated difficulties in professional functioning. Such an approach could facilitate the early identification of occupational situations characterized by increased stress and could direct preventively measures toward potentially modifiable organizational factors. The principles of this approach are consistent with the literature highlighting the usefulness of interventions targeting work organization and the work climate for reducing occupational burnout and improving staff well-being [45,56].

The importance of integrated monitoring of occupational factors is also supported by objective data on absenteeism recorded at the same institution, which revealed significant variations in absences associated with respiratory infections, including COVID-19, among healthcare personnel [57]. These findings suggest the value of correlating occupational health indicators with data on work organization and professional functioning to obtain a more complete picture of the impact of professional demands on staff.

At the same time, the literature highlights the limitations of interventions focused exclusively on strengthening individual resilience, in the absence of structural changes to working conditions and workload. Such interventions are considered insufficient both from the perspective of healthcare personnel and based on available empirical evidence [12,58]. Organizational and team resources can help reduce the impact of occupational demands, but they cannot fully offset the effects of persistent overwork when it is not addressed at the organizational and systemic levels [14].

### 4.7. Strengths of the Study

One of the study’s main methodological strengths is the use of a convergent mixed-methods design, which allowed for the integration of quantitative results with the participants’ subjective experiences, expressed qualitatively, within the same sample. This approach made it possible to examine the phenomenon both through the lens of statistical associations between occupational factors and indicators of professional functioning, and through an analysis of how these experiences were perceived and described by the staff. The sample size (n = 903) is substantial for a single-center occupational medicine study, and the inclusion of multiple professional categories provided a broader perspective on occupational experience than that offered by studies focused exclusively on physicians or nurses’ categories that frequently predominate in the literature [59,60].

### 4.8. Limitations of the Study

This study has a number of limitations that should be taken into account when interpreting the results. The instrument used is not a validated questionnaire for assessing burnout, and the research did not aim to measure or estimate the prevalence of burnout as a clinical or occupational entity defined by a standardized instrument. This limitation is part of a broader methodological issue in the field, characterized by the heterogeneity of the instruments and criteria used to assess burnout [33,60]. Furthermore, the cross-sectional nature of the study and the retrospective, self-reported collection of professional experiences do not allow for the establishment of temporal or causal relationships between the analyzed factors and the outcome variables; consequently, the results must be interpreted exclusively in terms of the observed associations.

Data collection took place in 2024, while the occupational experiences assessed (workload changes, relocation, sector assignment, and associated distress) largely pertained to the earlier COVID-19 pandemic period (from March 2020 onward, i.e., a recall window of up to approximately four years); this retrospective, single time-point assessment introduces the possibility of recall bias, whereby the accuracy of reported experiences may be affected by the time elapsed since they occurred and by subsequent events. Recall may also have been differential, as respondents currently experiencing greater distress may recall the pandemic period more negatively, which could inflate the observed associations.

Only participants with complete data were included in the primary models, and excluded participants differed systematically from those included (shorter length of service, less frequent COVID-sector assignment, and lower frequencies of the outcomes), indicating that data were probably not missing completely at random. We addressed this through multiple imputation of the missing workload, age, and tenure values (Section 2.4 and Section 3.2); results were materially consistent for most associations, with the exception of the workload association with physical/mental fatigue, which attenuated to borderline significance. This imputation procedure was implemented as a custom routine (bootstrap-based multinomial logistic and linear regression imputation models combined using Rubin’s rules) rather than a validated software package, and should be regarded as an approximate, illustrative sensitivity analysis rather than a definitive correction for non-random missingness. The two infection-restricted models (difficulty returning to work and difficulty with night shifts) retain a limited events-per-variable ratio despite the collapsed occupational-category variable; Firth penalized regression produced estimates close to the standard models for both outcomes (Section 3.2), suggesting that sparse-data bias was not a major driver of these results, though their wider confidence intervals still warrant cautious interpretation.

The qualitative component was analyzed collaboratively by two researchers, without an initial stage of independent coding and, consequently, without the possibility of calculating a formal measure of inter-rater agreement. Thematic saturation was not formally assessed, and no independent recoding of a subset of responses was performed. Response engagement with the open-ended item differed substantially by sector (90.9% vs. 40.8%; Section 3.3), and the between-sector comparison of theme frequency in the full sample (Table 4) is therefore confounded, to an extent that varies by theme, with this differential engagement; the restricted-sample sensitivity analysis (Table 5, Section 4.3) should be considered alongside the full-sample results when interpreting these themes. In addition, recruiting participants during routine medical checkups may introduce a potential health worker/attendance bias, as staff members absent from the routine evaluation were not represented in the sample. The complete flow of eligibility, participation, and exclusion of potentially eligible individuals could not be fully reconstructed. Finally, the single-center nature of the study limits the generalizability of the results to other healthcare institutions, other categories of staff, or different organizational and regional contexts.

### 4.9. Future Research Directions

Future research should include, in addition to the assessment of occupational and organizational factors, validated instruments for measuring burnout, psychological distress, and occupational resources, in order to directly test the relationships between job demands, available resources, and outcomes related to health and occupational functioning. A longitudinal design would help clarify the temporal sequence and direction of the identified associations, which is particularly relevant in light of recent data suggesting the possibility of bidirectional relationships between indicators of exhaustion and work capacity [61].

The rigor of the qualitative component could be strengthened by using independent raters, developing an explicit coding framework, and formally assessing inter-rater agreement. Furthermore, a systematic investigation of the relationship between high job demands and the positive dimensions of professional experience—such as a sense of usefulness, job satisfaction, and the perceived meaning of work—could contribute to a more nuanced understanding of the mechanisms of occupational adaptation and resilience among healthcare personnel in the context of a health crisis.

## 5. Conclusions

Among the study population, perceived workload and organizational disruption associated with staff relocation or redeployment were the occupational factors most consistently associated with impaired professional functioning. In contrast, simple assignment to a sector with increased infectious risk did not present significant independent associations with four of the five outcome variables analyzed, suggesting that the intensity of work demands and organizational changes in work activity may be more relevant to professional functioning than the administrative criterion of the sector of activity for most outcomes; an exception, discussed with appropriate caution, was a lower likelihood of difficulty returning to work among infected COVID-sector staff.

The qualitative analysis complemented these results by identifying specific occupational demands overwork, fear of infection, adaptation to new roles, and difficulties associated with the use of personal protective equipment as well as perceived occupational resources, in particular peer support and job satisfaction. Organizing these themes within Job Demands–Resources framework allowed for a coherent integration of quantitative and qualitative data and highlighted the convergence between the structured indicators and experiences reported by participants.

From an occupational medicine perspective, the results support the importance of directly monitoring workload and organizational changes that may affect professional functioning, as relevant elements for assessing psychosocial risks and guiding preventive measures. Team resources identified through qualitive analysis may represent potential directions for intervention; however, their protective role requires confirmation through further studies, preferably longitudinal and based on validated instruments.

## Figures and Tables

**Table 1:** Characteristics of the cohort (n = 903).

Characteristic	Value
Female sex, n (%)	802 (88.8%)
Age, mean ± SD	47.76 ± 8.55 years
Length of service, mean ± SD	18.10 ± 10.34 years
Nurse, n (%)	416 (46.0%)
Auxiliary care staff, n (%)	212 (23.5%)
Physician, n (%)	106 (11.7%)
Resident physician, n (%)	66 (7.3%)
Administrative staff, n (%)	45 (5.0%)
Other occupational categories, n (%)	58 (6.4%)
Workload—unchanged, n (%)	222 (24.6%)
Workload—slight increase, n (%)	288 (31.9%)
Workload—significant increase, n (%)	276 (30.6%)
Workload—missing or invalid, n (%)	117 (13.0%)
Staff relocation/redeployment, n (%)	259 (28.7%)
Work in a COVID-19 designated unit, n (%)	253 (28.0%)

SD: standard deviation. Workload was missing or invalid for 117 participants (116 unanswered items and one respondent who selected two contradictory categories).

**Table 2:** Occupational predictors of distress and functional impairment—full multivariable logistic regression models (all covariates).

Outcome (n; Events)	Predictor	aOR	95% CI	*p*	q (FDR)
Major stress (n = 781; 430 events)	Slight workload increase	0.84	0.59–1.21	0.354	—
	Significant workload increase	1.31	0.89–1.93	0.178	0.222
	Age (per year)	1.01	0.99–1.04	0.216	—
	Years of service (per year)	1.00	0.98–1.02	0.942	—
	Female sex	1.12	0.71–1.77	0.629	—
	Work in COVID sector	0.72	0.49–1.05	0.090	0.135
	Relocation	1.70	1.19–2.41	0.003	0.007
	Physician/resident physician	1.06	0.67–1.67	0.812	—
	Other staff	0.85	0.60–1.21	0.378	—
Physical/mental fatigue (n = 781; 566 events)	Slight workload increase	1.69	1.14–2.49	0.009	—
	Significant workload increase	2.04	1.31–3.17	0.001	0.004
	Age (per year)	1.03	1.00–1.05	0.034	—
	Years of service (per year)	1.01	0.98–1.03	0.512	—
	Female sex	1.04	0.63–1.72	0.872	—
	Work in COVID sector	1.09	0.70–1.71	0.703	0.801
	Relocation	2.16	1.41–3.32	<0.001	0.002
	Physician/resident physician	1.13	0.67–1.92	0.646	—
	Other staff	0.87	0.59–1.29	0.491	—
Difficulty maintaining work pace (n = 781; 128 events)	Slight workload increase	1.98	1.01–3.87	0.046	—
	Significant workload increase	7.16	3.80–13.49	<0.001	<0.001
	Age (per year)	1.04	1.01–1.08	0.011	—
	Years of service (per year)	1.02	0.99–1.05	0.146	—
	Female sex	0.65	0.34–1.23	0.183	—
	Work in COVID sector	0.94	0.58–1.53	0.801	0.801
	Relocation	1.64	1.04–2.61	0.035	0.058
	Physician/resident physician	1.08	0.58–2.01	0.796	—
	Other staff	0.62	0.36–1.05	0.075	—
Difficulty returning to work [infected subgroup] (n = 562; 58 events)	Slight workload increase	1.76	0.81–3.81	0.153	—
	Significant workload increase	3.94	1.82–8.51	<0.001	0.002
	Age (per year)	1.03	0.99–1.08	0.179	—
	Years of service (per year)	1.04	1.00–1.08	0.043	—
	Female sex	0.46	0.21–1.01	0.053	—
	Work in COVID sector	0.38	0.17–0.86	0.020	0.037
	Relocation	0.89	0.44–1.83	0.756	0.801
	Physician/resident physician	1.02	0.42–2.45	0.967	—
	Other staff	0.64	0.29–1.39	0.259	—
Difficulty with night shifts [infected subgroup] (n = 562; 65 events)	Slight workload increase	2.45	1.10–5.46	0.028	—
	Significant workload increase	4.43	2.00–9.85	<0.001	0.002
	Age (per year)	1.05	1.00–1.10	0.038	—
	Years of service (per year)	1.01	0.97–1.05	0.625	—
	Female sex	0.52	0.23–1.21	0.131	—
	Work in COVID sector	0.58	0.30–1.14	0.113	0.155
	Relocation	2.49	1.35–4.57	0.003	0.007
	Physician/resident physician	0.93	0.43–1.97	0.843	—
	Other staff	0.18	0.07–0.45	<0.001	—

aOR: adjusted odds ratio; CI: confidence interval; q: Benjamini–Hochberg FDR-adjusted *p*-value, computed jointly across the 15 primary coefficients of interest (significant workload increase, relocation, and COVID-sector assignment, for each of the five outcomes) and shown only for these coefficients. Reference categories: unchanged workload; nurse. Difficulty returning to work and difficulty with night shifts were analyzed among participants with a documented SARS-CoV-2 infection.

**Table 3:** Distribution of emerging themes identified in responses to the open-ended question (n = 903).

Category	Theme	n (%)
Strain/outcome	T1—Emotional stress and exhaustion	195 (21.6%)
Job Demands	T2—Fear of infection/concern for family safety	134 (14.8%)
Job Demands	T3—Workload and overwork	114 (12.6%)
Job Demands	T4—Adaptation to new roles	123 (13.6%)
Job Demands	T5—Issues related to personal protective equipment (PPE)	46 (5.1%)
Job Resources	T6—Peer support	43 (4.8%)
Job Resources	T7—Professional fulfillment	15 (1.7%)

Each response could be classified under one or more themes simultaneously; therefore, percentages are not mutually exclusive and do not sum to 100%.

**Table 4:** Frequency of emerging themes—COVID-19 unit staff vs. remaining staff (Fisher’s exact test; Benjamini–Hochberg FDR correction).

Theme	COVID-19 Unit Staff (%)	Remaining Staff (%)	OR	95% CI	*p*	q (FDR)
T5—Issues related to PPE	17.8	0.2	140.41	19.24–1024.91	<0.001	<0.001
T4—Adaptation to new roles	28.9	7.7	4.87	3.27–7.23	<0.001	<0.001
T6—Peer support	10.3	2.6	4.26	2.27–8.01	<0.001	<0.001
T7—Professional fulfillment	5.1	0.3	17.55	3.93–78.35	<0.001	<0.001
T3—Workload and overwork	20.2	9.7	2.35	1.57–3.52	<0.001	<0.001
T2—Fear of infection	22.1	12.0	2.08	1.43–3.05	<0.001	<0.001
T1—Emotional stress and exhaustion	26.5	19.7	1.47	1.05–2.06	0.031	0.031

CI: confidence interval; FDR: false discovery rate. Columns 2 and 3 show the percentage of respondents within each group (COVID-19 unit staff, n = 253; remaining staff, n = 650) who reported the corresponding theme. Because only 495 of 903 participants (54.8%) answered the open-ended item, and response rates differed markedly by sector (Section 3.3), these percentages reflect both the frequency of each theme and the frequency of answering the item; a sensitivity analysis restricted to respondents who answered is presented immediately below.

**Table 5:** Theme frequency by sector, restricted to the 495 respondents who answered the open-ended item.

Theme	COVID Sector (%)	Remaining Staff (%)	OR (95% CI)	*p*	q (FDR)
T5—Personal Protective Equipment (PPE)	19.6	0.4	64.22 (8.77–470.03)	<0.001	<0.001
T7—Professional fulfillment	5.7	0.8	7.88 (1.76–35.29)	0.002	0.004
T4—Adaptation to new roles	31.7	18.9	2.00 (1.32–3.03)	0.001	0.003
T6—Peer support	11.3	6.4	1.86 (0.98–3.52)	0.057	0.080
T3—Workload/overwork	22.2	23.8	0.91 (0.60–1.39)	0.748	0.748
T2—Fear of infection	24.3	29.4	0.77 (0.52–1.15)	0.224	0.262
T1—Emotional stress/exhaustion	29.1	48.3	0.44 (0.30–0.64)	<0.001	<0.001

CI: confidence interval; FDR: false discovery rate. Rows are ordered by effect size, as in Table 4, to facilitate comparison.

**Table 6:** Correspondence between self-reported occupational factors and spontaneously reported qualitative themes.

Occupational Factor (Self-Reported)	Theme Present Among Exposed (%)	Theme Present Among Non-Exposed (%)	OR (95% CI)	*p*
Substantial increase in workload → Theme “Work overload”	29.7	5.3	7.56 (4.75–12.05)	<0.001
Staff redeployment/reallocation → Theme “Adaptation to new roles”	33.6	5.6	8.54 (5.59–13.05)	<0.001
Assignment to a COVID-19 designated unit → Theme “PPE-related discomfort”	17.8	0.2	140.41 (19.24–1024.91)	<0.001
Major perceived stress → Theme “Emotional stress/exhaustion”	30.5	12.5	3.06 (2.17–4.32)	<0.001

For each occupational factor, the corresponding qualitative theme was selected a priori on conceptual grounds. Odds ratios (OR) with 95% confidence intervals were calculated using Haldane–Anscombe correction where applicable; *p*-values were derived from Fisher’s exact test. PPE: personal protective equipment. The workload pair excluded participants with missing or invalid workload data (n = 786).

**Table 7:** Joint display: integration of quantitative and qualitative findings within the Job Demands–Resources framework.

Quantitative Factor	Qualitative Theme	OR	Illustrative Quotation	Interpretation (JD-R)
Increased workload	Work overload (T3)	7.56	“During COVID it was non-stop work and alertness, we barely had time to eat.”	Direct job demand; convergence consistent with conceptual overlap between measures
Staff redeployment	Adaptation to new roles (T4)	8.54	“I was moved to a different unit, with different patients, and had to learn everything on the spot.”	Organizational demand; convergence consistent with conceptual overlap between measures
Assignment to COVID-19 unit	PPE-related discomfort (T5)	140.41	“The protective suit was suffocating; I could barely breathe after a few hours.”	Specific physical demand; strongest convergence observed
Major perceived stress	Emotional stress/exhaustion (T1)	3.06	“I couldn’t rest between shifts, it was constant, prolonged tension.”	Strain outcome; moderate convergence
No direct quantitative counterpart	Peer support (T6)	—	“We supported each other, the team was extraordinary.”	Perceived job resource; disproportionately reported among the most exposed staff
No direct quantitative counterpart	Professional fulfillment (T7)	—	“I felt I could help, that I was needed—even though it was hard.”	Possible strain–meaning coexistence within a small subgroup

Rows 1–4 correspond to the four factor–theme pairs formally tested in Table 6, with odds ratios reported for the corresponding factor–theme associations. Rows 5–6 present the two resource-related themes, for which no directly corresponding quantitative variable was available; these are reported descriptively. Quotations were selected as illustrative examples and have been translated from the original Romanian.

## Data Availability

The dataset analyzed in this study contains potentially identifying information at the level of small occupational subgroups within a single institution and was collected under an ethics approval that did not include provisions for public deposition of individual-level data. A minimal anonymized/aggregated dataset sufficient to verify the statistical analyses reported in this manuscript is available from the corresponding author upon reasonable request, subject to institutional data-sharing requirements.

## Abbreviations

The following abbreviations are used in this manuscript: aOR: Adjusted Odds Ratio; CI: Confidence Interval; COVID-19: Coronavirus Disease 2019; FDR: False Discovery Rate; ICD-11: International Classification of Diseases, 11th Revision; JD-R: Job Demands–Resources (framework); OR: Odds Ratio; PPE: Personal Protective Equipment; q: FDR-adjusted probability value; SARS-CoV-2: Severe Acute Respiratory Syndrome Coronavirus 2; SD: Standard Deviation; SPSS: Statistical Package for the Social Sciences; WHO: World Health Organization.
